# To Stay or to Leave? Migrant Workers' Decisions During Urban Village Redevelopment in Hangzhou, China

**DOI:** 10.3389/fpubh.2021.782251

**Published:** 2021-11-08

**Authors:** Xizan Jin, Tianzhou Ren, Nuannuan Mao, Lili Chen

**Affiliations:** School of Management, Zhejiang University of Technology, Hangzhou, China

**Keywords:** migrant worker, housing demolition, urban village redevelopment, migration decision, housing choice

## Abstract

As a vital source of the demographic dividend, migrant workers living in urban villages have positively contributed to urban economic development and the improvement of urbanization. Although urban villages have had a great impact on public health due to the shabby environments and poor public safety, the large-scale demolition of the urban villages, the supply of affordable housing for migrant workers has decreased drastically, which may lead to the outflow of many migrant workers and consequently affects the sustainable operations of cities. Therefore, this paper takes Hangzhou as an example to study the impact of urban village redevelopment on migrant workers and their migration decisions during urban village redevelopment process. The finding indicates that migrant workers are significantly impacted by large-scale demolition. (1) The number of affected migrant workers is huge. For example, 657,000 migrant workers who lived in around 178 urban villages are affected in Hangzhou (34,468 households). (2) The increase in rent is obvious. (3) Strong expulsion effect: nearly 1/3 migrant workers will decide to leave the city because of the demolition. Furthermore, our binary logistic regression model suggests that the commuting time, living satisfactory, and the rent affordability are factors significantly affecting migration workers' decision to leave and stay in the city. The housing quality and comfort indicators are not significant. This indicates that convenience for employment and high rent avoidance are the major characteristics of migrant workers' housing choice. Hence, in addition to considering whether the harsh environment is harmful to the public health of urban and residents, the interest and characteristics of migrant workers should be considered during the current urban village demolition process. While simply demolishing urban villages, government needs to provide a relatively sufficient amount of low-cost and affordable housing for migrant workers in case migrant workers leave the city in large numbers due to lack of suitable housing in the city.

## Introduction

Urbanvillages have been caused by China's unique land ownership system and the accelerating process of urbanization (1–5). Most of them are disparaged as neighborhoods of moldy housing, garbage-strewn streets, poor public safety, and unplanned land use due to poor environmental quality and high crime rates, which have a large negative impact on public health (6–8). Therefore, urban villages are generally recognized as undesirable enclaves and demolition is supported by the authority in many cities (9–11).

In spite of the urban villages have negative effects on public health, they provide a suitable housing market for migrant population (8, 12). On the one hand, affordable housing is available for low-income migrant workers by informal renting contracts with local villagers. Without urban villages, those migrants are unable to access to rental housing in the open market by their low income (3, 13). On the other hand, urban villages serve as a buffer zone for rural migrants to adjust to urban life (14). Urban villages not only ease migrants' stress of surviving in the metropolis with low living cost (15, 16), but also relieve their mental pressure caused by their incompatibility when they first entered the metropolis from the countryside (14). The urban village is a new community mode for migrants to settle, and work (10, 17).

The transformation and sustainable development of China's social economy cannot be separated from the support of the group of migrant workers (14, 18). As an important part of economic society, migrants work plays multiple roles in urbanization and industrialization (19, 20). From the production point of view, migrants work is equal to labor migration, which are engaged in various industries. For local governments, an adequate influx of labor can ease the contradiction of urban labor, effectively reduce labor costs, and improve the efficiency of labor resource allocation, which has positively contributed to the growth of economy (20). From the perspective of living conditions, local authorities should provide basic living resources for the incoming migrant work, such as alternative and affordable housing levels. Therefore, many scholars have suggested that in the short term, the urban village is a pragmatic and effective solution in providing affordable housing for rural migrants, and deserves moderate tolerance with regard to problems caused by its existence (21–23).

The demolition of urban villages helps to improve the living standards of permanent urban residents, thus promoting long-term sustainable and healthy economic development, but on the other hand, it will lead to mass expulsions of rural migrants (24). In addition, due to the limited housing opportunities for low-income migrants, they keep facing the reality that their chances to improve their housing and access to jobs by moving have not improved as a result of the redevelopment of urban villages (25). This part of migrant workers can only continue to cluster in urban villages or low-rent housing in the edge of the city (26), which cannot fundamentally solve public health problems arising from the urban villages before.

Therefore, to a certain extent, the problem of urban villages has become one of the significant issues in the process of Chinese urbanization. The question of how to demolish and build urban villages, which reflects a highly complex social value orientation and development standards, has received attention from the academic community and from observers with different backgrounds. In recent years, a number of scholars have conducted extensive research on the causes of urban villages (17, 27), redevelopment modes (28, 29), distribution of interests and compensation (30, 31), conflicts in land collection (32, 33), and financing methods (34, 35) from different perspectives. However, most early scholarly works on urban villages focus on the villagers, government, and real estate developers (17, 32, 36), with limited attention to migrant workers.

But with in-depth understanding of urban villages and rethinking on its redevelopment in recent years, a larger number of scholars have gradually realized that focusing only on the resettlement of local villagers while neglecting the large number of migrant workers is unscientific. It is agreed that the purpose of urban village redevelopment is to effectively create value for all stakeholders. As a mega project, the redevelopment can improve the environment, as well as way of current life and production when the interests of all parties are considered (37, 38). As a vital source of the demographic dividend, migrant workers living in urban villages have positively contributed to urban economic development and the improvement of urbanization. However, they are unable to settle down and enjoy the urban life during the process of transformation. Thus, the demand of this group should be considered so that each social class can benefit from urban development, reflecting the true meaning of urbanization (38, 39). It has been admitted that a residential environment can influence the human capital accumulation of labor from multiple channels (40, 41). The urban village redevelopment would be unfair and ineffective if it is realized at the price of sacrificing the interest of residential environment of migrant workers in urban villages (37, 38). As one of the ways to share the fruits of urbanization, a pattern of urban village redevelopment that benefits migrant workers will accelerate the urbanization process, resolve the conflict between supply and demand of affordable housing, and prevent new problems (38, 42). Therefore, protecting the interests of migrant workers during the urban village redevelopment is significant. However, to solve this issue, several questions should be clarified, such as the effect of demolition on migrant workers, how these workers migrate, and what are the factors that affect these migrations. Recent scholarly works do not address these questions clearly.

This study selects Hangzhou as the research object, which has suffered from large-scale demolition in recent years. Different from the previous studies from the perspectives of villagers, governments, and real estate developers. This article focuses on migrant workers who live in urban villages. And this study contributes by exploring the significant effects of the large-scale urban village demolition and the main factors in the selection of migratory decision (migrants' leave or stay) in order to provides theoretical and practical references for urban village redevelopment policy given the benefits of the migrant workers.

## Migration Decision of Migrant Workers During Redevelopment of Urban Villages in Hangzhou

### Emergence and Development of Urban Villages in China

Urbanization is a necessary process and stage for the development of a country and society. Since the 1980s, China's urbanization process has further accelerated, the rate of which reached 58.52% in 2017 according to national statistics. However, as China's industrialization and urbanization process continues to accelerate, the demand for land by capital, technology, and other production factors is increasing, making limited urban land resources scarcer. It is inevitably that the city converts sub-urban rural land for urban use (2, 43, 44). In this process, the farmland will be requisitioned rather than the rural settlement to avoid compensation cost and time-consuming relocation of indigenous villagers (17). Consequently, for a long time, the rural settlement gradually became spatially surrounded or annexed by new urban development, leading to the formation of urban villages. And meanwhile, the rapid development of urban construction and industries absorbed a large number of migrant workers, which created an increasing demand for housing with low rent. Due to the low income or the need for saving, migrant workers cannot afford or unwilling to pay all the regular rental housing prices (3, 13, 17, 45). Due to the less regulated and lack of standardization, local residents in urban villages provide low-rent housing for migrant workers with informal contracts (21, 46). Obviously, urban villages are a realistic and efficient urban housing market for the migrants.

Although low-rent urban villages have become the most important place of residence for migrant workers, there is the lack of legal protection of the leasehold due to the insecure property rights and villagers are less motivated to maintain their building and improve neighborhood environment. In addition, the gathering of low-income migrant workers has brought about many problems such as social problems, and environmental damage. Most of the urban villages are often associated with dirty, overcrowding, garbage-strewn streets, unplanned land use, and more social problems such as crimes, fire hazards, and con?icts, even China's “slum,” which have restricted the urbanization and sustainable development of cities (17, 47, 48). These are the primary reasons why many cities support the demolition of urban villages (9). Many local governments believe that urban villages are in need for demolition and redevelopment which can not only promote the efficient use of land resources but also contribute to the public economy sustained healthy development of the city (24, 49). And the faster the process of industrialization and urbanization of the city is, the larger the scale of urban villages redevelopment.

### Urban Village Redevelopment and Influence in Hangzhou

Because of rapid urbanization, Hangzhou has started its urban village redevelopment since 1998. According to “Implementation Suggestions on Launching the 5-year Critical Action about Urban Villages Reconstruction in the Main District of Hangzhou (2016–2020),” (hereafter “The Plan”) which was jointly issued by the Hangzhou Municipal Committee and Municipal General Office, there were 246 urban villages in total, and 68 were planned to be completed before the end of 2015. If the inspection of these 68 urban villages fails to meet the standards, the redevelopment should be continued, thereby ensuring that standards are met by the end of 2017. In addition, according to the plan, 178 villages will continue to be redeveloped next 5 years. One hundred 39 of them will be demolished, 21 of them will be regenerated which the work mainly focuses on refurbishing the outer facades of buildings, to improve the environment and reflect village's history and culture, 18 of them will combination of the two mode. Which means in the urban village, partial area would be demolished, while other area would be regenerated.

In order to further study the specific impact of urban villages redevelopment, we investigated a large number of urban villages and interviewed many relevant managers and villagers. We found that this large-scale centralized redevelopment has had a great impact on society, especially migrant workers:

First, the size of the affected population is enormous. A great number of migrant workers live in urban villages. Based on the survey conducted, the number of migrant workers in an urban village is 9–10 times larger than that of local residents, as shown in Table 1^1^. For example, the Wulian Community in Xihu District consists of 2,800 local residents and 573 houses, while there were 27,840 migrant workers; Luojiazhuang consists of 2,642 local villagers, 526 households, and ~30,000 migrant workers; and Guantangcun consists of ~2,500 local people, 516 households, but ~20,000 migrant workers. According to the statistics about tenants suggested by the Public Security Department on August 28, 2017, ~657,000 migrant workers lived in 178 urban villages, accounting for 17.8% of the main urban population. These statistics imply that nearly 2 out of every 10 inhabitants will be affected by the redevelopment.

**Table 1:** The number of the local residents and migrant workers in urban village we surveyed.

	**Wulian community [Xihu (WestLake) district]**	**Zongguantang community (Gongshu district)**	**Dongguan community (Binjiang district)**	**Huafeng community (Xiacheng district)**	**Sanjiaocun community (Yuhang district)**
Local residents	About 2,800	About 2,500	About 2,000	About 3,110	About 4,982
Migrant works rent in urban village	About 27,840	About 22,800	About 16,000	About 25,000	About 42,000

Second, the increase in the rental costs of migrant workers is obvious. Due to the large-scale and centralized demolition, the supply of affordable rental housing for migrant workers is drastically dwindling while the demand is rapidly increasing. According to Baidu index of renting in cities, Hangzhou's index of renting in 2017 was 70.48% greater than that in the previous year, which was significantly higher than the rates in Shanghai, Beijing, and Nanjing. Furthermore, the supply shortage speeds up the increase in rental fee. For instance, the monthly rent in Luojiazhuang and Wulian community was 700–800 yuan per room in 2016. In the following year, it increased by more than 50% to around 1,200–1,500 yuan per room, and the rent per square meter was as high as 100 yuan. This is also proved by the rents of the urban village change we surveyed during centralized urban village redevelopment, as shown in Table 2.

**Table 2:** The rents of the urban village change we surveyed during centralized urban village redevelopment.

	**Wulian community [Xihu (WestLake) district]**	**Zongguantang community (Gongshu district)**	**Dongguan community (Binjiang district)**	**Huafeng community (Xiacheng district)**	**Sanjiaocun community (Yuhang district)**
Original rent ¥	700–800¥	600–700¥	700–800¥	800¥	600–700¥
After rent ¥	1,200–1,500¥	1,000–1,200¥	1,300–1,400¥	1,200¥	1,100–1,300¥

The rising rents not only increase the living cost of migrant workers, but also increase employment cost of labor-intensive enterprises. For example, based on our survey, Lvjing, a representative Cleaning Company in Hangzhou, reflected that its employees' salary was increased by 300–500 yuan (about 20%) per month in 2017, which means that the management pressure of labor-intensive enterprises has further heightened. From the microeconomic view, migrant workers' decision on staying in the city or leaving is impacted by the expected income and expected cost in the urban. The expected income includes the salary and social benefit. The expected costs include living expenses and housing cost, whether it is for renting or buying. Due to the rise in rents, the living expenses of migrant workers have greatly increased, so they can only ask for a raise or seek higher-paying jobs, which can ensure that they can survive in the city.

Third, anxiety has been prevailing with the shortage in rental housing. Because of the large-size demolition, not only migrant workers but also residents of 18,000 households needed rental housing, thereby causing a shortage of rental housing in the market. The housing shortage adds to migrant workers' anxiety and insecurity as they have to spend a considerable amount of time, efforts, and resources to find new settlements. The survey shows that migrant workers are highly dissatisfied with the urban village redevelopment.

Fourth, the social losses are increasing. Several migrant workers experience difficulty in continuing their small-scale operations in urban villages. Moreover, social and neighborhood relationships that have been formed will be broken up. Therefore, the migrant workers' sense of social integration and belonging will be adversely affected.

### Migration Decision of Migrant Workers During Demolition

As analyzed, the large-scale and centralized demolition has had a significant effect on the lives of migrant workers. The demolition of urban villages results in social exclusion and gentrification (50) and has also caused large numbers of low-income migrant workers lost the low-cost living communities that they once depended on (51). And after displacement, migrant workers also difficultly find suitable housing nearby, during large-scale centralized demolition in the city, which may keep them away from cities and workplaces (26, 52). Therefore, when an urban village is to be demolished, migrant workers will consider immigration decisions based on the expected income and expected cost of living in the city (whether to stay or leave the city).

(1) Returning to rural areas. It is evident that the high housing price has a “negative” impact on the migrants' decisions on settling down, as their living costs are increased not only directly by rental housing price but also indirectly by the increased sale price of goods due to the high lease cost of stores (53–55). With the increase in rent due to the demolition, some migrant workers decide to return to home village because they cannot adapt to the city life with the increased cost in all aspects. If once many migrant workers leave Hangzhou, this may lead to a shortage of labor, especially for labor-intensive companies in city. Because most migrant workers in urban villages engage in labor-intensive industries, and some of them work in labor-intensive companies as cleaners, security guards, construction workers, and others, which are indispensable to the city's sustainable development (56–58). Thus, if many of a great number of these migrant workers choose to leave the city due to the impact of the urban village demolition, it will be not only more difficult for these labor-intensive enterprises to recruit workers, but also it will compromise the city's service level and reduce the city's competitiveness, thereby affecting its operation capacity.
(2) Still try to stay in the city by renting housing with high price in the neighborhood such as commodity house, or selecting peasant houses in farther suburbs with low rent. The former involves the people at a good economic level and those who have to stay in this area, mainly because they have stable work here or their children study nearby. However, losing the low-cost living community on which they depend for survival and facing with the pressure of rising rents, they have to find other jobs with higher wages or require companies to raise wages, thereby putting further pressure on labor-intensive firms (59). But more people have no choice but to relocate to remote urban villages or suburban area (60), which may drive them further away from the city and places of work. In addition, the relatively high living cost of long-distance commute will be shifted to companies if migrant workers still work in the city center.

According to the data from our survey, when low-cost rental housing is demolished, 30% of the migrant workers suggest that they are consider to return to their home village, which reflects the phenomenon of a large number of migrants escaping from the city. The rationale is that, unlike the classical urban–rural push–pull theory of population migration, the current push–pull conditions have changed. From the point of view of the city, the transformation from high-income pull to high-cost push is evident. In the past, there was a tremendous pull by high incomes in city obviously, attracting people to come to the city for a better life. At present, however, the high-income pull is no longer competitive because the high-cost push due to the cost of living, such as renting or purchasing a house, is considerably high. And the high-income pull is gradually no match for the high-cost push.

But as studied by Liu (61) and Wei (62), China's migrant workers are always excluded from housing guarantee system, but migrant workers are an important factor affecting social stability. The government should improve the policy for migrant workers to enhance their sense of belonging to the city where they work and thus retain the labor force (63). Thus, in parallel with the background of large-scale urban village redevelopment, three questions should be explored: What type of migrant workers will choose to leave Hangzhou? What kind of living conditions and policies will affect their decision? What measures should be taken to reduce the negative effect during the demolition and to retain migrant workers?

## Empirical Research: What Influences Migration Decisions of Migrant Workers

### Sample and Data Collection

The data on the migration decisions choices of migrant workers were obtained from a questionnaire survey involving 500 migrant workers who mainly lived in urban villages. Questions included personal characteristics (such as gender, age, marriage, income, employment status, etc.), housing characteristics, housing expenditure, social housing support, and migration decisions.

Before questionnaire surveys, we conducted preliminary qualitative interviews, and on-site visits to understand the researched context better, which led to better research questions, variable selection, sampling, and questionnaire design. For example, our visits to local urban village community managers helped us understand the distribution of migrant population and housing rentals, which enabled us to focus on typical areas/migrant groups. Moreover, face-to-face interviews implemented by trained interviewers facilitated the understanding of questionnaire items and thus ensured better data quality. The data collection lasted for 60 days until the sample met the standard for analysis. First, we visited the Hangzhou Municipal Construction Commission (the municipal authority responsible for urban village redevelopment) to get a preliminary understanding of the urban village redevelopment situation in Hangzhou. Second, according to the start-up situation of urban villages provided by the construction committee, one urban village under demolition was selected and investigated in each district. These districts include: Xihu district, Gongshu district, Yuhang district, Binjiang district, and Xiacheng district. The local residents of urban villages investigated by us are shown in Table 3. As a result, 100 migrant workers were randomly chosen for data collection in each district.

**Table 3:** Sample distribution.

**Gender (%)**		**Age (%)**		**Marriage (%)**		**Average monthly income (%)**		**Commuting time (one way) (%)**		**Living satisfaction (%)**	
Male	54.6	≤ 30	40.6	Single	28.2	≤ 2,000	14.2	<15 min	46.5	Highly satisfactory	5.2
Female	45.4	31–45	31.6	Married	71.8	2,001–4,000	35.8	15–30 min	23.6	Partly satisfactory	14.8
		46–60	25.6			4,001–6,000	28.4	30–45 min	14.5	Satisfactory	43.8
		≥61	2.2			6,001–8,000	11.8	45–60 min	9.7	Unsatisfactory	28.2
						8,001–10,000	5.0	>60 min	5.7	Highly unsatisfactory	8.0
						≥10,001	4.8				
Total	100	Total	100	Total	100	Total	100	Total	100	Total	100

The sample distribution is presented in Table 4. Among our sample of 500 migrant workers, males account for 54.6% and females account for 45.4%. 40.6% of migrant workers are under 30 years old, 71.8% are married. The monthly income of these migrant workers is mostly between 2,000 and 6,000 yuan, accounting for 64.2%. As for their current residence, 46.5% of migrant workers live within a 15-min commute, but their satisfaction is low, with only 8% of them are highly satisfactory.

**Table 4:** The urban village we surveyed.

**District of the Hangzhou**	**WestLake district**	**Gongshu district**	**Binjiang district**	**Xiacheng district**	**Yuhang district**
Urban village	Wulian community	Zongguantang community	Dongguan Community	Huafeng community	Sanjiaocun community
Local residents	About 2,800	About 2,500	About 2,000	About 3,110	About 4,982
Total houses	573	588	713	860	991

*We adopt the principle of only one migrant worker per tenant (choose only one person in the same room)*.

### Model and Variable

According to the theory of population migration and existing research, individual, family, economic, social, and institutional factors, may push or pull migration and affect individual decision to stay or leave. As the most basic while the biggest expense of rural migrants to survive in cities, housing is the key factor impacting their decisions (64, 65). In order to further to explore the influence of housing in the urban villages on migrant workers' decisions and effectively promote housing supply for migrant workers, this study divides the migration decision during the process of urban village redevelopment into two categories: leaving Hangzhou and staying in Hangzhou (including staying away from the city center or staying in the original area). Since the dependent variable is a typical discrete variable, this paper builds the binary logistic model as follow:


(1) $$\begin{array}{l} Y=\beta_{0}+\beta_{1}X_{1}+\beta_{2}X_{2}+\ldots +\beta_{n}X_{n}+\varepsilon \end{array}$$

where Y is the migrant workers' decisions during the urban village redevelopment process, Y = 1 represents leaving Hangzhou, Y = 0 represents staying in Hangzhou; X_1_, X_2_……X_n_ are explanatory variables; β_1_, β_2_……β_*n*_ are regression coefficients of each explanatory variable; and ε is the random error. As this study focuses on the effect of the urban village housing redevelopment on migration decision, housing-related factors consist of housing expenditure and consumption capacity, physical characteristics of current rental [such as residential space, facilities, and location (commuting) status] (66, 67), and available social housing support. Current monthly rental expense and rent affordability are specific indicators to measure housing expenditure and consumption capacity. Current physical characteristics of current rental housing are measured by commuting time, per-capita rent area, private kitchen and bathroom, and living satisfaction. “Knowing the government's housing security policy for migrant workers” and “existence of a housing subsidy” are used as measurement indicators for social housing support. In addition, personal characteristics, such as gender, marriage, age, duration of stay in Hangzhou, average monthly income, and whether with a labor contract or not, are used as control variables. There variables are discrete, except the per-capita rent area which is a continuous variable. To make the value of variables comparable and keep the variable data in the interval [0,1], the data is discretely standardized as follows:


(2) $$\begin{array}{l} Z=\frac{X-minX}{maxX-minX} \end{array}$$

where Z is the standardized data; X is the raw data; minX is the minimum value in the data; maxX is the maximum value in the data.

The characteristics and descriptions of variables are detailed in Table 5.

**Table 5:** The characteristics and descriptions of variables.

	**Variable number**	**Variable** **label**	**Variable assignment**
Personal characteristics	Y	Choice	0 = stay, 1 = leave
	X_1_	Gender	0 = male; 1 = female
	X_2_	Marriage	0 = single; 1 = married
	X_3_	Age	1 = under 30; 2 = 31–45; 3 = 46–60; 4 = above 61
	X_4_	Duration of stay in Hangzhou	1 = within 1 year; 2 = 1–3 years; 3 = 3–5 years; 4 = more than 5 years
	X_5_	Average monthly income	1 = under 2,000 yuan; 2 = 2,001–4,000 yuan; 3 = 4,001–6,000 yuan; 4 = 6,001–8,000 yuan; 5 = 8,001–10,000 yuan; 6 = above 10,001 yuan
	X_6_	Whether the person signed a labor contract	0 = NO; 1 = YES
Housing characteristics	X_7_	Commuting time (one way)	1 = within 15 min; 2 = 15–30 min; 3 = 30–45 min; 4 = 45–60 min; 5 = more than 60 min
	X_8_	Per-capita rent area	Actual value
	X_9_	Private kitchen and bathroom	1 = with private kitchen and bathroom; 2 = with private bathroom, no kitchen; 3 = with private kitchen, no bathroom (as a reference)
	X_10_	Living satisfaction	1 = highly satisfactory; 2 = partly satisfactory; 3 = satisfactory; 4 = unsatisfactory; 5 = highly unsatisfactory
Housing expenditure	X_11_	Current monthly rental expense	1 = under 500 yuan; 2 = 501–1,000 yuan; 3 = 1,001–1,500 yuan; 4 = 1,501–2,000 yuan; 5 = above 2,001 yuan
	X_12_	Rent affordability	1 = under 500 yuan; 2 = 501–1,000 yuan; 3 = 1,001–1,500 yuan; 4 = 1,501–2,000 yuan; 5 = above 2,001 yuan
Social housing support	X_13_	Knowing the government's housing security policy for migrant workers	0 = NO; 1 = YES
	X_14_	Housing subsidy	0 = NO; 1 = YES

### Results and Analysis

To test and compare the influence of factors at different levels on migration decision, the binary logistic model is adopted to estimate several variables (Models 1–3) and all variables (Model 4). The results are shown in Table 6. Evidently, the more explanatory variables are considered, the higher is the accuracy of the result, and the estimated result of the coefficient is steady. Hosmer–Lemeshow tests also show that the models are valid. Therefore, an empirical analysis is conducted based on Model 4.

**Table 6:** Ordered logit regression results for factors influencing migrant workers' choice.

**Variable type**	**Variable name**	**Model 1**		**Model 2**		**Model 3**		**Model 4**	
		**B**	**Sig**.	**B**	**Sig**	**B**	**Sig**.	**B**	**Sig**.
Personal characteristics	Gender	−0.398*	0.067	−0.537**	0.022	−0.522**	0.027	−0.505**	0.034
	Marriage	1.256***	0.000	1.412***	0.000	1.415***	0.000	1.472***	0.000
	Age	0.073	0.599	0.037	0.808	0.009	0.956	0.033	0.835
	Duration of stay in Hangzhou	0.080	0.438	0.052	0.634	0.058	0.594	0.048	0.666
	Average monthly income	−0.309***	0.001	−0.326***	0.001	−0.323**	0.002	−0.329***	0.001
	Whether signed a labor contract	−0.278	0.207	−0.339	0.142	−0.346	0.137	−0.248	0.297
Housing characteristics	Commuting time (one way)			0.184**	0.037	0.178**	0.045	0.186**	0.038
	Per-capita rent area			−0.026	0.819	0.012	0.917	0.017	0.887
	Private kitchen and bathroom				0.793		0.865		0.827
	Private kitchen and bathroom (1)			−0.393	0.421	−0.307	0.535	−0.360	0.468
	Private kitchen and bathroom (1)			−0.310	0.529	−0.235	0.637	−0.288	0.566
	Private kitchen and bathroom (1)			−0.817	0.385	−0.731	0.447	−0.797	0.407
	Living satisfaction			0.426**	0.000	0.383**	0.002	0.385**	0.002
Housing expenditure	Current monthly rental expense					−0.010	0.905	−0.006	0.943
	Rent affordability					−0.250**	0.009	−0.243**	0.012
Social housing support	Knowing the government's housing security policy for migrant workers							−0.973**	0.026
	Housing subsidy							0.212	0.626
Model fitting and effect index	Nagelkerke *R*^2^	0.144		0.214		0.233		0.247	
	Hosmer–Lemeshow test	0.064		0.695		0.525		0.529	
	Forecast accuracy	0.668		0.718		0.735		0.728	

*indicates P ≦ 0.1, **indicates P ≦ 0.05, and *** indicates P ≦ 0.001.

The model shows, in the variables of housing characteristics, there is only variables such as commuting time and living satisfaction are significant at 5% level and the coefficient is 0.186 and 0.385, respectively. This result denotes that with longer commuting time and lower living satisfaction, migrant workers are more likely to decide to leave Hangzhou. However, the variables of per-capita housing area and private kitchen and bathroom have minimal influence on migrant workers' decisions. These two factors represent migrant workers' needs for living space, living quality, and comfort. For many migrant workers, the purpose of working in cities is mainly to earn more money, and the housing space just only meets their most basic living needs. Therefore, for migrant workers, the effect of improving housing area and comfort will not be significant.

And among the two variables of the housing expenditure, only rent affordability is significant (coefficient −0.243). Because relative to absolute rent, this indicator can measure the housing consumption capacity of the migrant workers more reasonably. Migrant workers allocate their limited income on the basis of their own conditions. Some of their income is allotted to housing expenses and other commodity consumption, and the rest is saved or is provided to their family. Once the rent is out of their expectation, the migrant workers' living quality and benefits in the city will decline sharply. Thus, the migrant workers are more likely to choose leaving Hangzhou because of “difficult to pay” or “unaffordable” cost of housing.

Overall, in the variables of housing characteristics and housing expenditure, commuting time, and rent affordability are key factors that influence the migration decision. In contrast, factors such as per-capita rent area and private kitchen and bathroom, which represent housing quality and comfort, are not crucial. This situation testifies the characteristics of employment trend and rent aversion when migrant workers choose housing. Employment trend means that migrant workers prefer to rent housing close to their workplace, for convenience of living near work and reducing commuting cost. Rent aversion refers to the fact that migrant workers' housing options are flexible to housing price while inelastic to the nature and quality of housing. Consequently, migrant workers can accept crowded and narrow housing for lower rental cost. In other words, if the interests of migrant workers and the characteristics of housing options are ignored during the urban village redevelopment process, and just simple demolition is adopted, but at the same time, no adequate alternative to low-cost housing supply is provided. It may cause migrant workers to decide to leave the city, which will lead to the outflow of labor and preventing the urban economy from moving forward.

In social housing support, the coefficient of knowing the government's housing security policy for migrant workers is −0.973, which is significant. In recent years, housing security policy has not been limited to local households, which means that migrant workers can also apply for public rental housing. Additionally, according to Hangzhou's point management of residence permits, as long as the years of renting housing reach the standard, the points in renting are equal to purchasing. By this way, tenants are able to enjoy their rights in the same way as buyers in many aspects, including compulsory education, public health, basic old-age pension, employment service, community affairs, science and technology declaration, housing security, and others. However, our survey suggests that these policies remain unnoticed by migrant workers, as only 19.1% of them expressed their understanding of these policies. Therefore, propaganda of related housing policies available for migrant workers should be strengthened.

As another variable in social housing support, housing subsidy is not significant for migrant decision, as the current housing subsidy in our sample might be relatively low. Our data suggests that migrant workers with housing subsidy only account for 7.6%. Since the influence coefficient is positive, however, the housing subsidy may ease migrant workers' pressure on housing costs to a certain extent and may also motivate them to stay in Hangzhou to a certain extent.

With regard to personal characteristics, the control variables of gender, marriage, and average monthly income have significant impacts on migration decision. (1) Gender is an effect variable with extraordinary significance. Women are more willing to stay in Hangzhou than men, which is similar to Zakharenko's (68) findings on international migration. The backflow probability of women is lower than that of men because of two main reasons. With the development of industrialization and the service industry, the demand for female labor has increased. Besides, in China, men are traditionally responsible for supporting their family and parents, which means that the pressure on women to earn money as well as to take care of elderly parents is relatively low. Therefore, the rising cost of living in the city and their old parents in the home village may drive men to leave Hangzhou. (2) Marriage, a significant variable with a coefficient of 1.472, shows that married people are more prone to returning to home village than single people. For married people, the cost of relocation (including currency, physiology, and psychology) is higher, especially for families with school-age children. Our survey indicates that plenty of interviewees have no choice but to go home because urban village demolition affects their children's education. (3) Average monthly income has a coefficient of −0.329, which is significant. This factor has a negative correlation with the probability of labor transfer, which is also consistent with the classical population theory.

## Conclusion and Insights

### Conclusion

With the background of large-scale urban village redevelopment in China, numerous scholars have analyzed this redevelopment process from the perspectives of villagers, governments, and developers, whereas only a few studies have been conducted based on migrant workers, who also play an important role in urban villages. Therefore, this paper takes Hangzhou as an example to explore the impact of urban village redevelopment on migrant workers and their migration decisions during this process. The finding indicates that migrant workers are significantly impacted by large-scale demolition. (1) The number of affected migrant workers is huge. For example, 675,000 migrant workers who lived in around 178 urban villages are affected in Hangzhou. (2) The increase in rent is obvious. (3) Strong expulsion effect: nearly 1/3 migrant workers will have to leave the city because of the demolition. Furthermore, the binary logistic regression model is adopted for the analysis of factors that influence the reported migration decision. The result suggests that the impacts of these factors, such as commuting time, living satisfaction, rent affordability, knowing the government's housing security policy for migrant workers, gender, marriage, and average monthly income, are significant.

### Insights

The socio-economic transformation of developing countries has led to rapid urbanization and the acceleration of rural-urban migration (69, 70). Therefore, several recommendations will be proposed according to the analysis, as reference for improving policies of urban village redevelopment and minimizing the negative effect of sharply decreased urban village housing on labor.

(1) *Regarding housing characteristics, only commuting time, living satisfaction, and rent affordability are key factors that influence the migration decision. By contrast, factors such as per-capita rent area and private kitchen and bathroom, which represent housing quality and comfort, are not crucial*. This result validates the characteristics of employment trend and rent aversion when migrant workers choose housing to a certain extent. In other words, to avail themselves of low rent and avoid long commutes, migrant workers can accept crowded and narrow housing spaces. Thus, the interest and housing choice feature of migrant workers should be considered during the current demolition, which means that adequate alternative low-cost housing should be provided while urban villages are being demolished. Three ways are available to supply sufficient alternative housing with low rent: (1) Based on industrial layout, and number and distribution of migrant workers, the government ought to plan and build collective dormitories or rental apartments specifically for migrant workers in all industrial and development zones, or in places where migrant populations and factories are relatively concentrated. This solution can reduce their cost of commuting and high housing consumption. (2) Encouraging urban villages to provide a modern and standardizing housing leasing service for migrant workers to improve their living quality and satisfaction. (3) Migrant workers' housing demand and living cost should be considered to increase the supply of rental housing that suits their needs. The rental housing ought to meet the standards of economic applicability, reasonable layout, scientific design, and guaranteed quality. In addition, the government is expected to support the site selection, land supply, and construction of other facilities to improve the habitability of housing areas.
(2) *Improvement of housing security policy for migrant workers*. Our study proves that knowing the government's housing security policy for migrant workers has a significant effect on their decision to stay, which demonstrates that housing security policy plays a positive role in preventing migrant workers from leaving the city. However, according to our survey, these policies remain unnoticed by migrant workers, as only 19.1% of them expressed their understanding of these policies. Therefore, the government should strengthen the propaganda to make migrant workers understand the content and implications of related housing policies and solve their housing problems in Hangzhou. Furthermore, the scope of housing security policies should be expanded. For instance, these policies can consider migrant workers through credit. Based on stable occupation and duration of stay and combined with the residential credit system, a graded empowerment mechanism for migrant workers to enjoy basic public services, such as housing security, should be gradually established.
(3) *Increasing the income level of migrant workers and solving the education problem of their children*. According to our study, the control variables, such as average monthly income, gender, and marriage, are also relevant factors. In addition, income inequality has a direct or indirect negative impact on public health (71, 72). Effective policies are necessary to increase migrant workers' income by improving their professional skills. As for married migrant workers, especially families with school-age children, ensuring equal rights to education is another crucial issue.

## Data Availability Statement

The raw data supporting the conclusions of this article will be made available by the authors, without undue reservation.

## Author Contributions

TR: data curation and formal analysis. NM and LC: investigation. XJ, NM, and LC: methodology. XJ: writing original draft. XJ and TR: writing—review and editing. All authors contributed to the article and approved the submitted version.

## Funding

This research was funded by the National Social Science Fund of China (No. 17CSH026).

## Conflict of Interest

The authors declare that the research was conducted in the absence of any commercial or financial relationships that could be construed as a potential conflict of interest.

## Publisher's Note

All claims expressed in this article are solely those of the authors and do not necessarily represent those of their affiliated organizations, or those of the publisher, the editors and the reviewers. Any product that may be evaluated in this article, or claim that may be made by its manufacturer, is not guaranteed or endorsed by the publisher.

## Footnotes

^1^Generally, the type of houses in Hangzhou urban villages can be divided into joint-user and multi-storey types, and the land size in the former is ~100 m^2^ with four storeys. Usually, the owner lives on the first floor and the other floors are divided into 5–6 rooms for rent, which can be up to 15–18 rooms. For the latter, the number of rental housing is less with 10 rooms per building.

## References

[1] Lin Y De Meulder B Cai X Hu H Lai Y. Linking social housing provision for rural migrants with the redevelopment of ‘villages in the city': a case study of Beijing. Cities. (2014) 40(Pt A):111–9. 10.1016/j.cities.2014.03.011

[2] Zhou ZH. Towards collaborative approach? Investigating the regeneration of urban village in Guangzhou, China. Habitat Int. (2014) 44:297–305. 10.1016/j.habitatint.2014.07.011

[3] Wu F Li LH Han SY. Social sustainability and redevelopment of urban villages in China: a case study of Guangzhou. Sustainability. (2018) 10:2116. 10.3390/su10072116

[4] Kochan D. Placing the urban village: a spatial perspective on the development process of urban villages in contemporary China. Int J Urban Region Res. (2015) 39:927–47. 10.1111/1468-2427.12287

[5] Yi ZY Liu GW Lang W Shrestha A Martek I. Strategic approaches to sustainable urban renewal in developing countries: a case study of Shenzhen, China. Sustainability. (2017) 9:1460. 10.3390/su9081460

[6] Zhu JM. From land use right to land development right: institutional change in China's urban development. Urban Stud. (2004) 41:1249–67. 10.1080/0042098042000214770

[7] Wang MF Lin XL Ning YM. Migrant populations, temporary residence, and urban village renovation: a survey of migrant settlements in Shanghai. City Plan Rev. (2012) 36:73–80. 10.11821/dlxb201508005

[8] Zhan Y. The urbanization of rural migrants and the making of urban villages in contemporary China. Urban Stud. (2018) 55:1525–40. 10.1177/0042098017716856

[9] Pan WJ Du J. Towards sustainable urban transition: a critical review of strategies and policies of urban village renewal in Shenzhen, China. Land Use Policy. (2021) 105744. 10.1016/j.landusepol.2021.105744

[10] Song Y Yves Z Ding CR. Let's not throw the baby out with the bath water: the role of urban villages in housing rural migrants in China. Urban Stud. (2008) 45:313–30. 10.1177/0042098007085965

[11] Lin SN Wu FL Li ZG. Social integration of migrants across Chinese neighbourhoods. Geoforum. (2020) 112:118–28. 10.1016/j.geoforum.2020.04.008

[12] Wu YZ Sun XF Sun LS Choguill CL. Optimizing the governance model of urban villages based on integration of inclusiveness and urban service boundary (USB): a Chinese case study. Cities. (2020) 96:102427. 10.1016/j.cities.2019.102427

[13] Zhu PY. Residential segregation and employment outcomes of rural migrant workers in China. Urban Stud. (2016) 53:1635–56. 10.1177/0042098015578614

[14] Du HM Li SM. Migrants, urban villages, and community sentiments: a case of Guangzhou, China. Asian Geographer. (2010) 27:93–108. 10.1080/10225706.2010.9684155

[15] Chen Y Dang YX Dong GP. An investigation of migrants' residential satisfaction in Beijing. Urban Stud. (2020) 57:563–82. 10.1177/0042098019836918

[16] Fan CC. The elite, the natives, and the outsiders: migration and labor market segmentation in urban China. Ann Assoc Am Geographers. (2002) 92:103–24. 10.1111/1467-8306.00282

[17] Hao P Sliuzas R Geertman S. The development and redevelopment of urban villages in Shenzhen. Habitat Int. (2011) 35:214–24. 10.1016/j.habitatint.2010.09.001

[18] Lin QY Mai Q. How to improve new generation migrant workers' entrepreneurial willingness—a moderated mediation examination from the sustainable perspective. Sustainability. (2018) 10:1578. 10.3390/su10051578

[19] He CF Chen TM Mao XY Zhou Y. Economic transition, urbanization and population redistribution in China. Habitat Int. (2016) 51:39–47. 10.1016/j.habitatint.2015.10.00610192561

[20] Luo JJ Zhang XL Wu YZ Shen JH Shen LY Xing XS. Urban land expansion and the floating population in China: for production or for living? Cities. (2018) 74:219–28. 10.1016/j.cities.2017.12.007

[21] Zhang L Zhao SXB Tian JP. Self-help in housing and Chengzhongcun in China's urbanization. Int J Urban Region Res. (2003) 27:912–37. 10.1111/j.0309-1317.2003.00491.x

[22] Wang YP Wang YL Wu JS. Urbanization and informal development in China: urban villages in Shenzhen. Int J Urban Region Res. (2009) 33:957–73. 10.1111/j.1468-2427.2009.00891.x

[23] Wu FL Zhang FZ Webster C. Informality and the development and demolition of urban villages in the Chinese peri-urban area. Urban Stud. (2013) 50:1919–34. 10.1177/0042098012466600

[24] Li M Xiong YH. Demolition of Chengzhongcun and social mobility of Migrant youth: a case study in Beijing. Eurasian Geography Econ. (2018) 59:204–23. 10.1080/15387216.2018.1503966

[25] Huang X Van Weesep J Tang SS. To move or not to move? Residential mobility of rural migrants in a medium-sized Chinese city: the case of Yangzhou. Housing Stud. (2021) 36:278–301. 10.1080/02673037.2019.1701634

[26] Liu Y Geertman S van Oort F Lin YL. Making the ‘invisible' visible: redevelopment-induced displacement of migrants in Shenzhen, China. Int J Urban Region Re. (2018) 42:483–99. 10.1111/1468-2427.12646

[27] Lin XB; Ma XG Li GC. Formation and governance of informality in urban village under the rapid urbanization process. Econ Geography. (2014) 34:162–8. 10.15957/j.cnki.jjdl.2014.06.016

[28] Yu H Zheng FT. The transformation model of urban villages: a case study of Hegezhuang, Beijing. Issues Agric Econ. (2011) 32:86–91. 10.13246/j.cnki.iae.2011.04.005

[29] Schoon S Altrock U. Conceded informality. Scopes of informal urban restructuring in the Pearl River Delta. Habitat Int. (2014) 43:214–20. 10.1016/j.habitatint.2014.03.007

[30] Hu Y Hooimeijer P Bolt G Sun DQ. Uneven compensation and relocation for displaced residents: the case of Nanjing. Habitat Int. (2015) 47:83–92. 10.1016/j.habitatint.2015.01.016

[31] Wang JM Liu MD Liu BH. Construction and verifying of benefit equilibrium model in urban village reconstruction. Areal Res Dev. (2015) 34:69–75. 10.3969/j.issn.1003-2363.2015.06.013

[32] Han HY Shu XF Ye XY. Conflicts and regional culture: the general features and cultural background of illegitimate housing demolition in China. Habitat Int. (2018) 75:67–77. 10.1016/j.habitatint.2018.04.008

[33] Ma XG Wang AM Yan XP. A study on land use conflicts in the urban spatial reconstruction process ——a case study of Guangzhou City. Hum Geography. (2010) 25:72–7. 10.13959/j.issn.1003-2398.2010.03.016

[34] Yang YP Song B. An empirical study on urban village redevelopment based on trust financing. J Commer Econ. (2010) 2:142–3. 10.3969/j.issn.1002-5863.2010.02.070

[35] Jiang YH Pang YS Han SS. Urban village reconstruction under the view of project management. J Civil Eng Manag. (2015) 32:59–66. 10.13579/j.cnki.2095-0985.2015.04.010

[36] Lai YN Tang BS. Institutional barriers to redevelopment of urban villages in China: a transaction cost perspective. Land Use Policy. (2016) 58:482–90. 10.1016/j.landusepol.2016.08.009

[37] Jia SH Zheng WJ Tian CH. Stakeholders' interest governance in the redevelopment of urban village: theories and countermeasures. City Plan Rev. (2011) 35:62–8.

[38] Fang J. Research of the Housing Problems Considering the Floating Population of Urban Village Reconstruction in Taiyuan City. Shanxi University of Finance and Economics (2014).

[39] Bao HJ Ye QY. The humanistic scale and welfare balance in the reconstruction of urban village based on Sen.'s capability theory. China Land Sci. (2015) 29:25–31. 10.11994/zgtdkx.2015.11.004

[40] Liu HY. Promoting human capital of the new generation of migrant workers by living integration. J Capital Univers Econ Bus. (2013) 5:77–81. 10.13504/j.cnki.issn1008-2700.2013.05.003

[41] Liu R Wong TC. Urban village redevelopment in Beijing: the state-dominated formalization of informal housing. Cities. (2018) 72:160–72. 10.1016/j.cities.2017.08.008

[42] Chen KS Tai H. Demand of floating people: a case of Wuhe, Bantian, Yangmei in Shenzhen. Modern Urban Res. (2015) 7:113–8. 10.3969/j.issn.1009-6000.2015.07.019

[43] Liu MQ Fu C. Commentary on domestic research literature in urban villages. Urban Insight. (2010) 6:177–85. 10.3969/j.issn.1674-7178.2010.06.020

[44] Liu YT He SJ Wu FL Webster C. Urban villages under China's rapid urbanization: unregulated assets and transitional neighbourhoods. Habitat Int. (2010) 34:135–44. 10.1016/j.habitatint.2009.08.003

[45] Zeng H Yu XF Zhang JF. Urban village demolition, migrant workers' rental costs and housing choices: evidence from Hangzhou, China. Cities. (2019) 94:70–9. 10.1016/j.cities.2019.05.029

[46] Yang SY van Oostrum M. The self-governing redevelopment approach of Maquanying: incremental socio-spatial transformation in one of Beijing's urban villages. Habitat Int. (2020) 104:102235. 10.1016/j.habitatint.2020.102235

[47] Wu FL. State dominance in urban redevelopment: beyond gentrification in urban China. Urban Affairs Rev. (2015) 52:631–58. 10.1177/1078087415612930

[48] Ding CR Qiu AJ Wang J. Migrant housing during rapid urbanization in China: typology and assessment. Urban Stud. (2011) 18:49–54. 10.3969/j.issn.1006-3862.2011.06.009

[49] Liang XW Yuan QF Tan XH Li ZG. Territorialization of urban villages in China: the case of Guangzhou. Habitat International. (2018) 78:41–50. 10.1016/j.habitatint.2018.05.009

[50] Qian JX He SJ Liu L. Aestheticisation, rent-seeking, and rural gentrification amidst China's rapid urbanisation: the case of Xiaozhou village, Guangzhou. J Rural Stud. (2013) 32:331–45. 10.1016/j.jrurstud.2013.08.002

[51] Ding K. Resettlement of floating population in the transformation of urban villages in Zhengzhou. New Countryside Heilongjiang. (2014) 8:334. 10.3969/j.issn.1674-8409.2014.08.331

[52] Liu Y Geertman S Lin YL. Heterogeneity in displacement exposure of migrants in Shenzhen, China. J Ethnic Migration Stud. (2018) 44:2562–81. 10.1080/1369183X.2017.1391078

[53] Costello L. Urban-rural migration: housing availability and affordability. Austral Geographer. (2009) 40:219–33. 10.1080/00049180902974776

[54] Michaelides M. The effect of local ties, wages, and housing costs on migration decisions. J Socio Econ. (2011) 40:132–40. 10.1016/j.socec.2011.01.010

[55] Liu WT. The influence of housing characteristics on rural migrants' living condition in Beijing Fengtai District. HBRC J. (2015) 11:252–63. 10.1016/j.hbrcj.2014.02.005

[56] Li BQ. Floating population or urban citizens? Status, social provision and circumstances of rural-urban migrants in China. Soc Policy Administr. (2006) 40:174–95. 10.1111/j.1467-9515.2006.00483.x

[57] Ma WL Chen W. Investigation and policy suggestions of housing conditions of rural migrant workers in Hangzhou. City Plan Rev. (2008) 32:38–44. 10.3321/j.issn:1002-1329.2008.05.006

[58] Zheng SQ Long FJ Fan CC Gu YZ. Urban villages in China: a 2008 survey of migrant settlements in Beijing. Eurasian Geography Econ. (2009) 50:425–46. 10.2747/1539-7216.50.4.425

[59] Ding XX. Housing prices, employee turnover and industrial upgrading. Market Weekly. (2018) 7:19–20.

[60] Logan JR Fang YP Zhang ZX. Access to housing in urban China. Int J Urban Region Res. (2009) 33:914–35. 10.1111/j.1468-2427.2009.00848.x 23976818 PMC3748629

[61] Liu SL. Analysis and countermeasures of urban housing security for migrant workers. Econ Manag Res. (2010) 1:50–55. 10.13502/j.cnki.issn1000-7636.2010.01.020

[62] Wei W. Housing affordability and its influencing factors of migrant workers in cities, a case study of Shanghai. Urban Prob. (2015) 11:98–103. 10.13239/j.bjsshkxy.cswt.151114

[63] Zhang JW. Analysis of the housing policy of migrant workers from the perspective of welfare analysis. Modern Bus Trade Industry. (2016) 14:37. 10.19311/j.cnki.1672-3198.2016.14.072

[64] Huang Z Deng XG. Analysis on the housing and integration of migrant workers into cities. Acad J Jingchu. (2013) 14:32–36. 10.14151/j.cnki.jcxk.2013.05.013

[65] Chen C Feng CC. Research on migrant workers' housing conditions and their willingness to stay in cities. Reform Econ Syst. (2011) 1:145–9.

[66] Zhao WH. Housing pressure and residence willingness: an empirical analysis on college graduates without registered residence in Beijing. Jiangsu Soc Sci. (2018) 2:31–40. 10.13858/j.cnki.cn32-1312/c.2018.02.006

[67] Sun C Song ZD Zheng SJ. The characteristics of migrant workers' housing demand and the optimization of urban housing security system——a study based on the investigation of urban village in Beijing. J Agrotech Econ. (2017) 4:16–27. 10.13246/j.cnki.jae.2017.04.002

[68] Zakharenko R. Return migration: an empirical investigation. SSRN Electron Journal. (2009). 10.2139/ssrn.1350375

[69] Yue ZS Wang BW Li SZ Fong E Feldman MW. Advantages of being bicultural: acculturation and mental health among rural-urban migrants in China. Cities. (2021) 119:103357. 10.1016/j.cities.2021.103357

[70] Tian L Yao ZH Fan CJ Zhou L. A systems approach to enabling affordable housing for migrants through upgrading Chengzhongcun: a case of Xiamen. Cities. (2020) 105:102186. 10.1016/j.cities.2018.11.017

[71] Hao Y Liu JH Lu ZN Shi RJ Wu HT. Impact of income inequality and fiscal decentralization on public health: evidence from China. Econ Model. (2021) 94:934–44. 10.1016/j.econmod.2020.02.034

[72] Jutz R. The role of income inequality and social policies on income-related health inequalities in Europe. Int J Equity Health. (2015) 14:117. 10.1186/s12939-015-0247-y 26521027 PMC4628290

